# En-bloc spondylectomy in the lumbar spine: indications, results and complications in a series of 47 patients affected by primary malignant bone tumors

**DOI:** 10.1007/s00402-024-05274-w

**Published:** 2024-04-08

**Authors:** Alessandro Luzzati, Stefano Conti, Isabella Sperduti, Alessandra Scotto Di Uccio, Simone Mazzoli, Luca Cannavò, Gennaro Scotto, Carmine Zoccali

**Affiliations:** 1 Oncological and Reconstructive Surgery Unit, IRCCS-Galeazzi Orthopedic Institute, Via Riccardo Galeazzi 4, 20161 Milan, Italy; 2 Department of Anatomical, Histological, Forensic Medicine and Orthopaedic Science, University of Rome, Piazzale Aldo Moro 5, 00185 Rome, Italy; 3 Statistical Department, IRCCS-Regina Elena National Cancer Institute, Via Elio Chianesi 53, 00144 Rome, Italy; 4 General Surgery and Organ Transplantation Unit, School of General Surgery, Umberto I Polyclinic of Rome, Sapienza University of Rome, Rome, Italy; 5 Oncological Orthopedics Department, IRCCS-Regina Elena National Cancer Institute, Via Elio Chianesi 53, 00144 Rome, Italy

**Keywords:** En-bloc spondylectomy, Wide surgery, Lumbar spine, Bone tumors, Wide margin, Spine surgery

## Abstract

**Introduction:**

Wide Surgery is the reference treatment for malignant and aggressive benign primary bone tumors in the spine. When located in the lumbar spine, En-Bloc Spondylectomy (EBS) remains a complex challenge. Moreover, surgery is complicated by the presence of the diaphragm in the thoracolumbar junction and the hinderance of the iliac wings at the lumbosacral levels. Therefore, EBS in the lumbar spine frequently requires combined approaches.

The purpose of this study is to describe clinical presentation, tumor characteristics and results of a series of 47 consecutive patients affected by malignant primary bone tumors of the lumbar spine who underwent EBS.

**Materials and methods:**

47 patients were reviewed. Complications were distinguished in early and late whether they occurred before or after 30 days from surgery.

Overall survival (OS), disease-free survival (DFS) and local recurrence-free survival (LRFS) were calculated by the Kaplan–Meier product-limit method from surgery until relapse or death.

**Results:**

27 patients presented to observation after a first intralesional approach in a non-specialized center. Chordoma was the most represented histotype. Vertebrectomies were: 23 one-level, 10 two-level, 12 three-level and 2 four-level. Reconstructions were always carried out with screws and rods. The main postoperative complication was blood loss, while hardware failure was the main long-term complication.

The 5-year LRFS was 75.5%, the 5-year DFS was 54.3%, and 5-year OS was 63.6%.

**Conclusions:**

The surgical margin obtained during the index surgery was statistically associated with Local Recurrence, DFS and OS, underlining the importance of treating patients in reference centers.

## Introduction

Although the spine is the most frequent site for bone metastases, primary benign aggressive and malignant bone tumors are uncommon. Therapy is usually based on the combination of chemotherapy, radiotherapy and surgery, with different schedules depending on the specific histologies.

Wide Surgery (WS) is the referral surgical treatment for malignant tumors in the spine, as in other sites as the limbs and pelvis. A marginal margin is considered acceptable in limited areas such as on the dura.

It may also be a viable alternative to reduce the risk of local recurrence in cases of benign but aggressive histologies [1]. WS could also be considered in metastatic long-survivors, mainly represented by patients with a solitary metastasis from favorable histologies, onset several years after the extirpation of the primary tumor [2, 3].

When WS consists in the removal of the entire vertebral body with negative margins, it is named En-Bloc Spondylectomy (EBS); the posterior arc is removed separately, in order to extract the vertebral body rotating it around the spinal cord to maintain the integrity of the nervous tissue [4].

Because of the increased potential of complications, EBS must be regarded as a very difficult procedure and should only be carried out by specialized teams who received training in it [5].

When the tumor is located in the lumbar spine, EBS remains a complex challenge. The proximity of the vertebrae to the retroperitoneal area increases the risk of damaging major neurovascular bundles. Moreover, tumors located in the thoracolumbar junction present surgical difficulties related to the presence of the diaphragm; whereas, when tumors are located in the lumbosacral junction, the presence of iliac wings can limit the access to the L4–S1 levels.

As a result, EBS in the lumbar spine frequently requires a combined anterior–posterior operation, whereas in the thoracic spine it is frequently accomplished utilizing a completely posterior approach. For these reasons, there are not any pertinent statistics on outcomes and complications because the literature only comprises brief series, case reports, and technique descriptions.

The purpose of this study is to review the data of a series of 47 patients affected by malignant primary bone tumors located in the lumbar spine who underwent EBS, to describe clinical presentation, tumor characteristics, results, and complications associated to the surgical treatment, highlighting the unique issues related to the lumbar spinal site.

The research question was: What are the outcomes and complications associated with EBS in patients with malignant primary bone tumors located in the lumbar spine?

## Methods

### Study design

This is a descriptive case series that covers all patients who underwent en-bloc procedures for malignant bone tumors of the lumbar spine in an orthopedic research institution between February 1993 and December 2021.

### Inclusion criteria

Patients who underwent en-bloc procedures for malignant bone tumors of the lumbar spine, treated between February 1993 and December 2021.

### Exclusion criteria

Patients who underwent en-bloc procedures for benign but aggressive bone tumors; patients alive with a follow-up lower than 12 months.

### Data collection

The series included 47 consecutive patients, 31 males and 16 females.

Diagnosis was performed by experienced pathologist trained in muscular skeletal tumors on the specimen obtained by CT-guided trocar biopsy or minimally-invasive open biopsy; in case of patients already treated in non-specialized centers, the diagnosis was made on the material obtained during the first surgery, if available, otherwise a new biopsy was carried out.

**Fig. 1:**
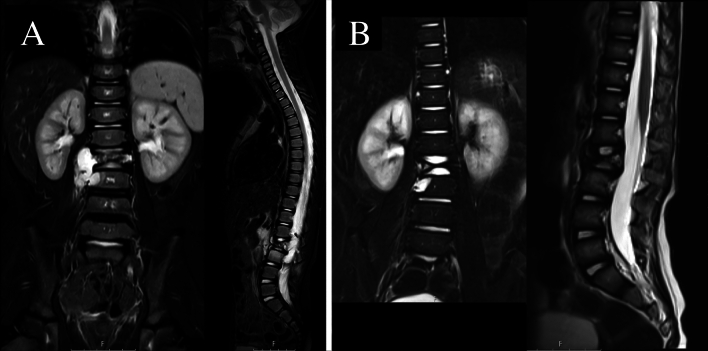
**a** MRI showing a 12-year-old male patient affected by a Ewing Sarcoma of L2-L3 vertebrae with intracanal compromission. **b** the same patient after neoadjuvant chemotherapy; the extraosseous component was no longer present

A full-body CT-scan was performed to rule out distant metastases. A spine MRI was also done to better visualize soft tissue components and to reveal possible intracanal elements (Fig. 1).

Angiography was always executed to locate the artery of Adamkiewicz; a selective embolization was also performed in case of hypervascularized tumors at risk of intralesional violation during surgery.

The Enneking staging system for malignant musculoskeletal tumors was used to assess the extent of the tumor [6].

The Weinstein-Boriani-Biagini System was applied to describe the vertebral areas compromised by the tumor and to assess the surgical strategy [7].

Surgeries were performed by the senior author, expert in muscular-skeletal spine surgery; antibiotic prophylaxis was administered based on the single case and its specific needs.

Patients were verticalized as soon as possible to decrease the risk of muscular atrophy and complications related to bed rest.

Complications were classified as early or late if they respectively happened before or after 30 days following surgery [8]. Blood loss was considered massive if it was more than the amount of blood that would normally circulate in 24 h or more than half that amount in 3 h. When the mean systemic pressure fell below 85 mmHg for more than 15 min, hypotension was declared. A worsening of the American Spine Injury Association (ASIA) score of at least 1 grade following surgery was deemed evidence of neurological impairment [9]. Diaphragm tears, pneumonia, and pulmonary embolism were reported as airway issues. Urinary retention and incontinence were urological issues.

The patients were followed-up every 3 months for the first 2 years, every 4 months during the third year, twice per year the fourth and fifth years and then, only in case of high-grade histologies, yearly for further 5 years.

Variations were possible based on specific histologies and problems.

### Statistical analysis

Descriptive statistics were used to summarize pertinent study information.

Median follow-up was estimated with the Reverse Kaplan–Meier method. Overall survival (OS), disease-free survival (DFS) and local recurrence-free survival (LRFS) were calculated by the Kaplan–Meier product-limit method from the date of the surgery until relapse or death. If a patient was not dead, survival was censored at the time of the last visit. DFS and LRFS were calculated from the date of surgery to the date of relapse or death. If a patient had not relapsed, DFS and LRFS were censored at the time of the last visit. The log-rank test was used to assess differences between subgroups. The hazard ratio, odds ratio and the confidence limits were estimated for each variable of interest outcome, using the Cox univariate model and logistic regression model, respectively. Significance was defined at the p < 0.05 level.

Gender, age, previous therapy, OR time, tumor volume, number of levels, blood loss, transfusions, hospital stay and radiotherapy were tested in univariate analysis for the early and late complication outcomes.

Gender, age, previous therapy, histology, margin, number of levels and tumor volume were tested in univariate analysis for the DFS, LRFS and OS outcomes.

A multivariate Cox proportional hazard model was also developed using stepwise regression (forward selection) with predictive variables which were significant in the univariate analyses. Enter limit and remove limit were p = 0.10 and p = 0.15 respectively. The SPSS (version 21.0; SPSS, Inc., Chicago, IL) licensed statistical program was used for all analyses.

All patients gave their consent to be included in the study and for its publication. This study received clearance from the institutional review board. It was designed in compliance with the Declaration of Helsinki’s guiding principles and the ethical guidelines of the relevant Committee on Human Experimentation.

Our research was reported using the STROBE statement for cohort studies.

## Results

### Epidemiology

Forty-seven patients, 31 males and 16 females, with an average age of 45 years (range 4–79 years, median 52) were enrolled in the study.

Specific histologies are reported in Table 1.

**Table 1:** Epidemiological and clinical characteristics

Parameters	Value
Patients	47 (31M–16F)
Age	Mean 45 years (range 4–79)
Sites	
TL	3 (6.4%)
L	38 (80.8%)
LS	6 (12.8%)
Enneking classification	
Intracompartimental	
IA	1 (2.1%)
IIA	3 (6.4%)
Extracompartimental	
IB	1 (2.1%)
IIB	41 (87.2%)
III	1 (2.1%)
Symptoms	
Pain	47 (100%)
Fracture	15 (31.9%)
Cauda equina syndrome	1 (2.1%)
Diagnosis	
Chordoma	16 (34.0%)
Ewing’s sarcoma	7 (14.9%)
Malignant peripheral nerve tumor	7 (14.9%)
Osteosarcoma	4 (8.5%)
Chondrosarcoma	4 (8.5%)
Myoepithelial carcinoma	2 (4.2%)
Leiomyosarcoma of bone	1 (2.1%)
Malignant hemangioendothelioma	1 (2.1%)
Paraspinal rhabdomyosarcoma	1 (2.1%)
Others malignant bone tumors	3 (6.4%)
Others malignant soft tissue	1 (2.1%)
Surgical approach	
Posterior	16 (34.0%)
Anterior–posterior	12 (25.5%)
Posterior–anterior–posterior	10 (21.3%)
Posterior–anterior	8 (17.0%)
Anterior–posterior–anterior	1 (2.1%)
Level of resection	
One-level	31 (66.0%)
Two-level	9 (19.1%)
Three-level	7 (14.9%)
Obtained surgical margin	
Wide	21 (44.7%)
Marginal	21 (44.7%)
Intralesional	5 (10.6%)
Intraoperative blood loss mean	Mean 4885 ml (range: 500–18000)

### Symptoms

All patients complained of pain, with 21 patients experiencing associated vertebral fractures. Based on the Asia Score, 33 patients were classified as “type E”, 13 as “type D”, and 1 as “type C”.

### Diagnosis

Diagnosis was performed after an average time of 6 months from the onset of symptoms (range 2–14 months, median 5).

Twenty-seven patients presented for observation after a previous erroneous approach in a non-specialized center.

Diagnosis was performed in three cases on the specimen obtained during the first inaccurate surgery, in 24 cases with minimally invasive biopsy and in 20 cases with CT-guided trocar biopsy.

### Sites and extension

Tumors were located in the lumbar spine in 38 cases, in the thoracolumbar junction in three cases and in the lumbosacral junction in six cases. They involved four levels in two cases, three levels in 12 cases, two levels in ten cases and one level in 23 cases (Table 1).

The average tumor size at diagnosis was 187 cm^3^ (range: 43–1020; median 100).

Out of 47 tumors, four were intracompartmental (one stage IA and three stage IIA) and forty-three were extracompartmental (one stage IB, 41 stage IIB, and one stage III) according to the Enneking staging system (Table 1).

The most prominent symptom was back pain, present in all instances and linked to a fracture in fifteen of them. Only one patient had Cauda Equina Syndrome at the time of diagnosis (associated to a vertebral fracture as well) (Table 1).

According to the Asia Score, one case was type C, twelve instances were type D, and thirty-four out of 47 patients were type E.

The tumor involved four sectors in only one instance, between five and nine sectors in 14 cases, and nine or more sectors in 32 cases, according to the WBB methodology. In 10 out of 47 instances, the spinal canal contained the tumor; 37 of these cases had extradural (“D” WBB tissue layers) and 9 had intradural (“E” WBB tissue layers) locations.

### Chemotherapy

Twenty-one patients received neoadjuvant chemotherapy, whereof eight also received adjuvant chemotherapy (Fig. 1). Chemotherapy was primarily reserved for high-grade chemo-sensitive tumors such as Ewing Sarcoma (seven cases, all of which were treated with neoadjuvant schedules, and three received adjuvant treatment as well), Malignant Peripheral Nerve Sheath Tumor (six out of eight cases, all of which were treated with neoadjuvant schedules, and three received adjuvant treatment as well), and Osteosarcoma (three out of four cases, all of which were treated with neoadjuvant schedules, and one received adjuvant treatment as well).

### Radiotherapy

Twenty-one patients underwent radiotherapy, whereof 15 before and 6 after surgery.

### Surgeries

The average number of involved levels was 2 (range: 1–4; median: 2).

The surgical approach was exclusively posterior in 16 cases, anterior–posterior in 12, posterior–anterior in 8, posterior–anterior–posterior in 10 and in only one case was anterior–posterior–anterior.

In one-level resections, the approaches were posterior in 12 cases (52.2%), posterior–anterior–posterior in six cases (26.1%), posterior–anterior and anterior–posterior in 3 (13%) and two cases (8.7%), respectively;

In two-level resections the approaches were anterior–posterior in six cases (60%), posterior and posterior–anterior–posterior in 3 (30%) and one case (10%), respectively;

In three-level resections the approaches were posterior–anterior in five cases (41.7%), anterior–posterior in three cases (25%), posterior–anterior–posterior in two cases (16.7%), posterior and anterior–posterior–anterior in one case each (8.3%);

In the two cases of four-level resection, the approach was anterior–posterior and posterior-anterior–posterior.

### Roots cut

Considering the entire series, the average number of roots cut for patients was 3.1, corresponding to an average value of 1.8 roots cut for each level.

### Reconstructions

**Fig. 2:**
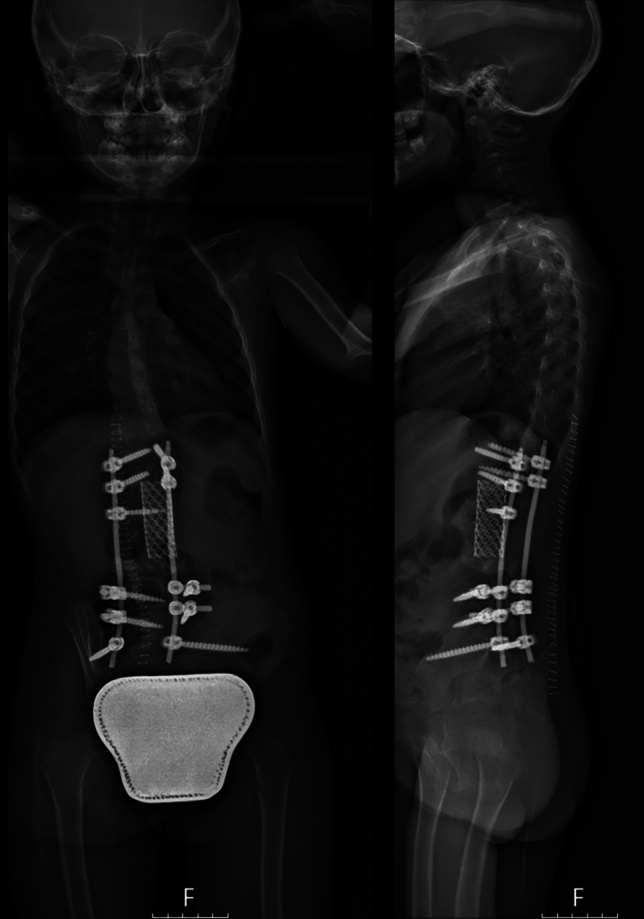
Post-operative X-ray showing posterior reconstruction with rods and screws; the anterior column was reconstructed with a titanium cage filled with autograft and homograft bone

All patients required posterior stabilization with rods and screws. 18 cases required anterior plating. The anterior column was rebuilt using an artificial cage in 12 cases, whereof seven made of carbon and five of titanium (Fig. 2); an iliac crest autograft was used in six cases; various techniques were used including cement, allografts, and fibular vascular autografts in the other cases.

### Blood loss and operation length

The average blood loss was 4885 ml (range 500–18000; median 4000) (Table 1). Eighteen patients required blood transfusions.

The procedures lasted around 10 h (average 590 min; median 600; range 180–900 min).

### Margins

Postoperative histologies revealed a wide margin in 11 cases, a marginal margin in 17 cases and an intralesional margin in 19 cases (Table 1). In all wide margin cases, the margin was planned wide; of the 17 marginal margin cases, eight were already planned as marginals, seven as wides but marginal on the dura and two wides with some intralesional transgression; in the 19 intralesional cases, the margin was predicted wide with some intralesional transgression in three cases, marginal in five cases and confirmed intralesional in 11 cases.

In the not previously treated and in the previously treated patients, the percentage of appropriate margins was 85.1% (12 marginal and five wide) and 74.1% (15 marginal and five wide), respectively; the difference is not statistically significant.

In 26 out of 47 instances (55%), the intended margin, preoperatively predicted by the surgeon, was verified at the final histology.

### Complications

Three patients died in the first postoperative months, whereof two for heart failure and one for disease progression with brain and lung metastases.

Early complications were reported in 27 out of 47 patients, whereof four experienced just one complication; the remaining part reported more than one complication.

The most common early complication was Massive Blood Loss (MBL) associated with hypotension, which occurred in 20 cases; in three patients it arose without hypotension; a case of hypotension not caused by MBL was also reported.

In 2 out of 20 cases of MBL associated with hypotension, a revision surgery was necessary.

Complications related to the spinal cord were also common: seven cases of dural tears were reported, whereof one with an associated meningocele which was surgically treated; two episodes of cord injury and one case of cauda equina syndrome resulted in a worsening of one Asia score grade in two cases.

A case of respiratory failure was reported as well.

No statistically significant association was found between patient gender, age, previous surgeries, surgery duration, tumor size, levels involved, hospital stay and radiotherapy and the onset of early complications; instead, blood loss and transfusions were statistically associated to early complications in univariate analysis (p = 0.002), whereas only transfusions maintained the association in multivariate analysis (p = 0.002).

Nineteen patients reported late complications. The most frequent was construct failure, that occurred in eight cases, whereof with and without loss of correction in six and two cases, respectively.

Deep wound infection was reported in four cases, whereof associated to dehiscence in one; superficial wound infection was present in two cases, both associated to dehiscences; All cases of wound infection were treated with surgical debridement and antibiotics. One case of Chylothorax resolved with conservative therapy was reported.

Overall, there have been five early surgical revisions, including two for hemorrhage and one for meningocele, and 12 late revisions, including six for wound infection and six for hardware failure resulting in loss of correction and stability.

A statistically significant association was found between the length of hospitalization and late complications; nevertheless, it is reasonable to sustain that late complications could cause a prolonged hospital stay.

Fifteen patients neither had early nor late complications.

### Follow-up

Considering the 23 living patients, the average follow-up is 64.2 months (range 12–190, median: 46).

### Recurrences

Data regarding recurrences is available for 44 cases. A total of eight recurrences occurred at 10, 15, 24, 24, 32, 42, 48, and 64 months from index surgery, whereof five in the 24 previously treated patients and three in 20 not previously treated patients; the difference is not statistically significant.

Related histologies were five out of eight Malignant Peripheral Nerve Sheath Tumors (62.5%), two out of 16 Chordomas (12.5%) and one of four Chondrosarcomas (25%).

**Fig. 3:**
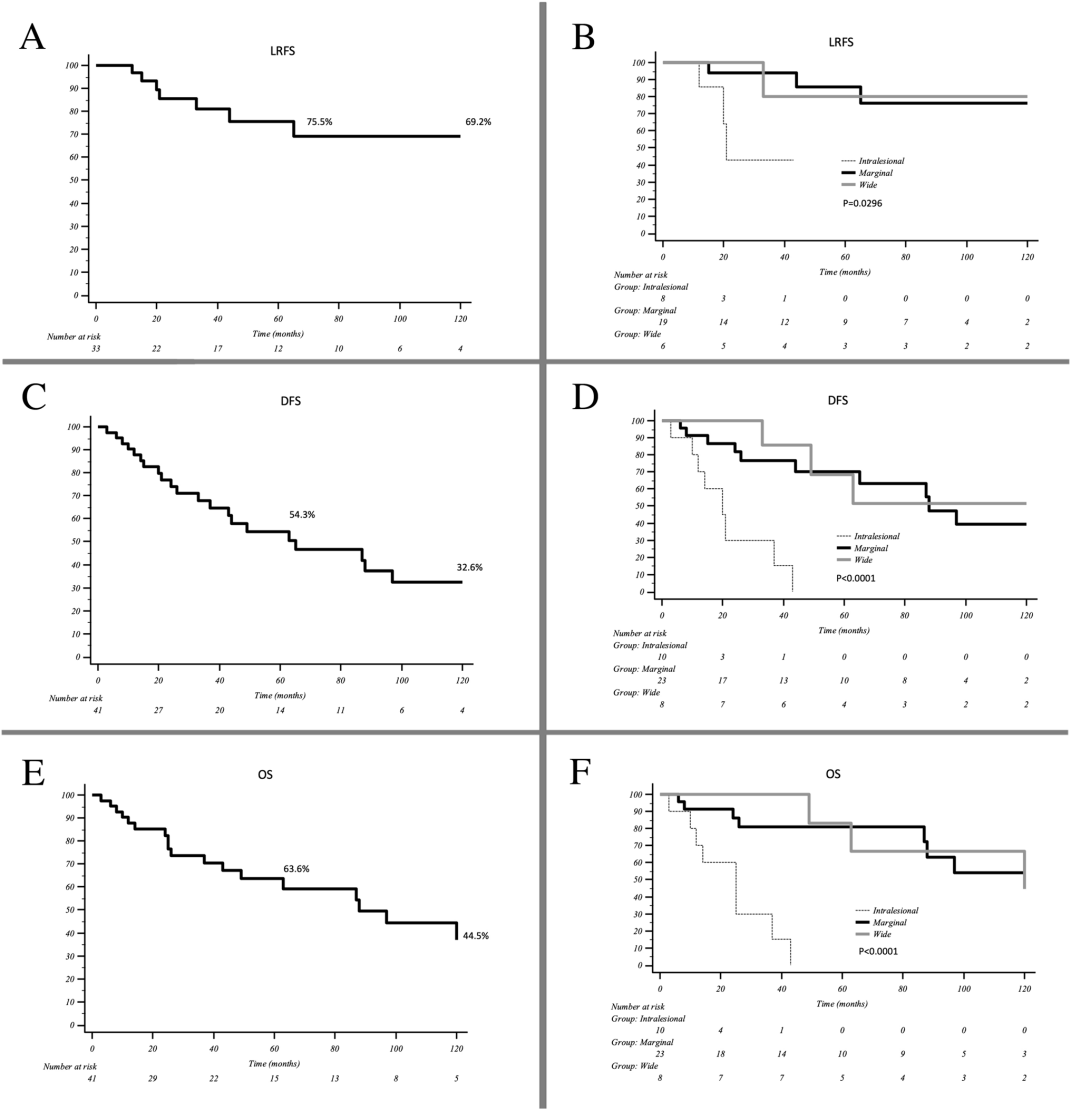
**a** the Kaplan Meier curve showing the risk of local recurrence and a 5y-LRFS of 75.5%; **b** the Kaplan–Meier curve showing LRFS related to the type of margin obtained during the first surgery. The difference between the adequate margin and the intralesional margin is statistically significant (p = 0.0296). **c** the Kaplan–Meier curve showing the DFS; the value at 5 years was 54.3%. **d** the Kaplan–Meier curve showing DFS related to the type of margin obtained during the first surgery. The difference between the adequate margin and the intralesional margin is statistically significant (p < 0.0001). **e** the Kaplan–Meier curve showing the Overall Survival; at 5 years, the survival rate is about the 63.6%. **f** the Kaplan–Meier curve showing the OS related to the type of margin obtained during the first surgery. The difference between the adequate margin and the intralesional margin is statistically significant (p < 0.0001)

The 5-year LRFS was 75.5%; the related Kaplan-Meir curve in shown in Fig. 3A;

No statistically significant differences are present between the previously treated and not previously treated patients.

LRFS results statistically different (p = 0.0296) according to the margin obtained during index surgery, as evident in the Kaplan Meier curve shown in Fig. 3B; while wide and marginal margins present a similar value, intralesional margins are strongly associated to a higher risk of local relapse (Table 2).

**Table 2:** The association between LRFS and the margin obtained during the index surgery

Local recurrence free survival	HR (CI95%)	p value
Pathology results	–	0.045
Intralesional vs wide	19.783 (1.364–286.964)	0.029
Marginal vs wide	2.558 (0.257–25.499)	0.423
Marginal vs intralesional	0.129 (0.020–0.817)	0.030

### Disease free survival

No statistically significant association was revealed between patient age, previous treatment, tumor histology and size; nevertheless, patient sex (p: 0.022) and margin obtained during surgery (p = 0.001), are statistically associated to DFS, both in univariate and multivariate analyses (Table 3).

**Table 3:** The association in univariate and multivariate analysis between patients’ characteristics and DFS; only the margin obtained during index surgery and gender resulted statistically associated in multivariate analysis

Disease-free survival	Univariate analysis		Multivariate analysis	
	HR (CI95%)	p value	HR (CI95%)	p value
Gender				
Male vs female	3.263 (1.189–8.949)	0.022	4.645 (1.505–14.335)	0.008
Age	1.010 (0.989–1.032)	0.338		ns
Previous therapy				
Yes vs no	1.086 (0.476–2.479)	0.844		ns
Histology	–	0.729		ns
Condrodarcoma vs malign tumor	1.468 (0.325–6.627)	0.617		
Cordoma vs malign tumor	0.793 (0.315–1.997)	0.622		
Cordoma vs condrosarcoma	0.540 (0.111–2.634)	0.446		
Patholgy results	–	0.001	–	< 0.0001
Intralesional vs wide	13.916 (2.993–64.703)	0.001	13.614 (2.871–64.559)	0.001
Marginal vs wide	1.956 (0.531–7.199)	0.313	1.015 (0.263–3.919)	0.98
Marginal vs intralesional	0.141 (0.046–0.432)	0.001	0.075 (0.20–0.282)	0.001
N. levels	1.132 (0.736–1.742)	0.573		ns
Tumor volume	1.001 (0.999–1.002)	0.580		ns

The 5-years DFS was 54.3%; the related Kaplan–Meier curve is reported in Fig. 3C.

Considering the DFS related to the margin obtained during the first surgery, the survival is different and shown in Fig. 3D. The differences between adequate and intralesional margins are statistically significant (p < 0.0001).

### Survival

At the last follow-up, 23 out of 44 patients were still alive, whereof 17 apparently free of disease, 4 with local recurrences, whereof 1 with distant metastases and 3 without, and 2 with distant metastases without local disease.

Regarding the 21 deceased patients, 6 died from unrelated causes and 15 died of disease, whereof 7 with local recurrence and 8 without.

The 5-years overall survival was 63.6%, the related Kaplan–Meier curve is reported in Fig. 3E.

The OS of patients who underwent resection with adequate margins and that of patients with intralesional margins resulted statistically different (p < 0.0001) (Fig. 3F).

No statistically significant correlations were found in univariate analysis between OS and patients’ gender, age, execution of previous surgeries, tumor size and volume whereas the margin was statistically significant (p < 0.0001) (Table 4).

**Table 4:** The association between the different items and the OS; the margin obtained during the index surgery is the only statistically significant

Overall survival	Univariate analysis	
	HR (CI95%)	p value
Gender		
Male vs female	1.939 (0.692–5.428)	0.208
Age	1.021 (0.995–1.047)	0.110
Previous therapy		
Yes vs no	1.053 (0.424–2.612)	0.912
Histology	–	0.486
Condrosarcoma vs malign tumor	1.954 (0.422–9.052)	0.392
Cordoma vs malign tumor	0.723 (0.265–1.972)	0.527
Cordoma vs condrosarcoma	0.370 (0.072–1.901)	0.234
Patholgy results	–	< 0.0001
Intralesional vs wide	14.423 (2.885–72.115)	0.001
Marginal vs wide	1.285 (0.336–4.908)	0.714
Marginal vs intralesional	0.089 (0.024–0.324)	< 0.0001
N. levels	1.081 (0.664–1.759)	0.755
Tumor volume	1.001 (0.999–1.002)	0.607

## Discussion

The study aimed to investigate the outcomes and complications associated with EBS in patients with malignant primary bone tumors situated in the lumbar spine. The majority of tumors were chordomas, followed by MPNSTs, Ewing’s sarcomas, chondrosarcomas, and osteosarcomas. The average age of patients was 45 years, with back pain being the most prevalent symptom.

As for every tumor, early diagnosis is important to increase survival. In the lumbar spine, pain is the main symptom; it is nonspecific and patients are often treated for degenerative problems for a long time before the tumor is identified.

Indeed, the average delay in diagnosis is quite consistent in the present series (6 months after onset of symptoms) and the disease was often identified when the tumor was already extracompartmental; moreover, patients are often initially managed in non-specialized centers that perform surgeries without a prior biopsy and specific histological diagnosis because scared by the compressive symptoms complained by the patient. In this scenario, the following salvage surgeries are technically more difficult and results are less satisfying than those in patients handled in referral centers [5].

Certainly, the previous surgery forces the surgeon to perform a more invasive and extensive surgery to try to decrease the risk of local recurrence.

Preoperative diagnosis is essential to choose the optimal strategy and histology is required to administer neoadjuvant chemotherapy, as for osteosarcomas and Ewing Sarcomas; moreover, several authors prefer to perform a longer and more intense neoadjuvant chemotherapy and then the extirpative surgery in the spine and pelvis, to avoid the risk of not performing the adjuvant treatment due to complications that are quite frequent in complex surgeries [10].

When possible, a transpedicular approach as minimally invasive as possible should be used to perform the biopsy.

Attention has to be paid for the L5 vertebra which presents pedicles inclined of about 45° on the sagittal axis, so the biopsy track will be longer and more difficult to remove en-bloc with the vertebra during index surgery so sometimes extra-pedicle biopsy also has to be considered.

Indeed, the CT-guided transpedicular trocar biopsy approach is characterized by minimal contamination; nevertheless, it may not be feasible in cases of extended necrotic tumors where the trocar biopsy may fail to obtain a sufficient specimen, causing significant delays in diagnosis. In such cases, a direct minimally invasive transpedicular approach could reduce the risk of sampling errors [11].

Surgery is the main treatment, often complemented with chemotherapy and radiotherapy for sensitive histologies [3, 12]. When surgeons must address primary tumors located in the lumbar spine, they must consider the specific anatomy and the close relationship with vascular bundles and nerve roots, which are responsible for the high rates of early and late complications.

Massive blood loss and neurological issues were the most common early complications, while construct failure and infections also occurred as late complications, emphasizing the importance of specialized surgical teams and preoperative planning to manage them.

Due to the proximity of lumbar vertebrae to vascular bundles, an anterior approach is often recommended, with only a minority of cases undergoing exclusive posterior approaches. Indeed, an exclusive posterior approach could be sufficient when the tumor is intracompartmental, but when it is extracompartmental and involves the great vessels, an anterior approach, such as lombotomic lateral extraperitoneal or anterior xypho-pubic, is strongly advised.

When the tumor is located in the thoracolumbar junction, EBS also involves diaphragmatic pillars, so surgeons may have to restore them by direct closure or by using a mesh when the diaphragm loss is more consistent [13]. When the tumor is located in the lumbosacral junction, EBS becomes very difficult due to the presence of the iliac wings and the sacroiliac joints; reconstructions can also be very difficult and involve the pelvis as well.

Other problems related to lumbar EBS are due to the resection of nerve roots. Indeed, while nerve sacrifice at the thoracic levels is mainly associated to local anesthesia, in the lumbar spine it is associated to important motor function loss, moreover when L3, L4, L5 or S1 roots are involved.

Actually, nerve roots can be sacrificed both because included in the tumoral mass and to remove the vertebrae in an easier and safer way.

Due to the intricate anatomy of this area, obtaining an adequate margin is difficult. In the spine, both wide and marginal margins are considered oncologically adequate, moreover in the dura area, whose removal to obtain a safer margin is only suggested in few cases [13].

The present study demonstrates that reaching an adequate margin is fundamental because it is the only factor statistically associated to the absence of local recurrence and to the OS. Oncological spine surgeons are forced to perform more invasive surgeries to resolve contamination associated to initial erroneous approaches [5].

Histological analysis revealed differing recurrence rates among tumor types; MPNSTs showed a very aggressive behavior, with a recurrence rate of 62.5% whereas Chordomas confirmed their lower aggressiveness, even more evident in the lumbar spine, with a recurrence rate of 12.5%.

Indeed, Chou et al., in 2017, reported a series of 29 primary MPNSTs with a recurrence rate of 48% [14]; the year before, the same group reported a recurrence rate of 35% in a series of 166 patients affected by Chordoma of the mobile spine [15].

According to an analysis of the Kaplan-Meyer curve, the risk of local recurrence plateaus after 48 months, however the first 4 years are thought to carry a significant risk of recurrence.

Complications are quite common in these surgeries: heart attack was the cause of the two postoperative surgery-related deaths. This underlines the impact of this kind of surgery on the patients.

Bleeding is one of the most important problems in these operations; the surgical team and the local transfusion center have to be aware of the possibility of consistent blood loss. Also, antibiotic prophylaxis should be tailored to maintain the blood levels.

Huang et al. reported a mean operative time and estimated blood loss of 282 min and 2421 ml, respectively, in a series of nine EBS procedures for metastasis, whereas Liljenqvist et al. obtained an average operative time of seven hours and an average intraoperative blood loss of 3210 ml [16, 17].

Massive blood loss is considered a major complication with a direct risk for the patient’s life, both for hemorrhagic shock and for disseminated intravascular coagulation and multiorgan failure.

Fisahn et al., in 2017, showed a higher risk of infections in patients who underwent spine surgeries, moreover in case of smokers [18].

The present paper confirms this data; blood loss and related transfusions were statistically associated to early complications.

Moreover, dural tears have to be considered possible complications, although the absence of the spinal cord distal to L2 decreases the risk of direct damage.

Infections are frequent late complications with consequent dehiscence of the wound; a precocious debridement is always suggested to decrease the risk of hardware contamination; indeed, while contaminated prostheses can be removed in the limbs, spine hardware cannot be replaced, and infection usually become chronic with the formation of fistulas; related sepsis can be considered a possible negative evolution.

Construct failure is the most common late complication and is often associated with loss of correction, necessitating further surgeries to maintain spinal equilibrium. Indeed, we reported revisions in 12.8% of cases at a follow-up of 64.2 months. Additionally, Shimizu et al. in 2018 published a paper on 30 patients who underwent TES for primary malignant and benign aggressive tumors of the lumbar spine, with a follow-up of 33 months. They reported a revision rate for instrumentation failure of 20.0% [19].

This rate increases in pediatric patients, with Luzzati et al. reporting a revision rate of 27.3% [12].

Hayashi et al. showed that a combined anterior and posterior approach is an independent risk factor for infection, suggesting the use of Iodine-supported spinal instrumentation to decrease the risk [20].

In the present series, the length of the hospital stay was directly associated to the onset of late complications. However, it could be considered a consequence and not a cause of the complications.

Reconstruction is an important topic after EBS. Indeed, the lumbar spine is quite mobile and a non-rigid fixation could cause precocious loosening. It has been observed that 40% of the EBS patients experienced late instrumentation failure, so a robust reconstruction is mandatory [21, 22].

Posteriorly, the number of levels involved in the arthrodesis is directly related to the extension of the resection. Although no guidelines are available, we suggest involving the pelvis and the distal thoracic levels.

Homoplastic/autoplastic grafts, titanium or carbon cages, or both, can be used for anterior column reconstruction; however, the hardware must ensure the excellent compression required for fusion, and given the significant stress forces acting on the lumbar spine and the torsion plane, the structure must be extremely stable to prevent mobilization [23].

Recurrence verified in eight cases (17% at an average follow-up of 64.2 months) with a 5y-LRFS of 78.9%; no difference was present between previously treated and not previously treated patients.

It is possible to hypothesized that reaching a wide margin at lumbar level could be important to compensate the supplementary risk caused by the first intralesional surgery.

Actually, it is possible that the specific histologies can influence the recurrence rate after salvage surgery in previously treated patients; less aggressive histologies as chordoma could be more easily resolved by an EBS.

The current rate was similar to those reported in the literature. Liljenqvist et al. reported two recurrences in a series of 11 patients (18.2%) who underwent EBS for primary tumors [17]. Shimizu et al. published an important series of 30 patients who underwent EBS for primary malignant and benign aggressive tumors of the lumbar spine, with a 33-month follow-up, reporting a recurrence rate of 13.3%. Additionally, the 10-year disease-free rate was 75.0% [19].

The global mortality rate is 45%; nine of the twenty-one patients who died had evidence of disease (eight with distant metastases, one with local recurrence) while the other twelve had no evidence of illness. The remaining twenty-six patients were still alive at the latest follow-up, whereof three with signs of local recurrence. At the most recent follow-up, no one in our cohort had systemic illness.

Malignant peripheral nerve sheath tumor (MPNST) was the most aggressive histotype, with high rates of recurrences, followed by chordoma and chondrosarcoma. In the eight patients affected by MPNST, recurrence rate was about 62.5%. Of the 16 patients affected by chordoma, recurrences were observed in only two patients, with a recurrence rate of about 12.5%; recurrences were observed in one of four chondrosarcomas (25%).

This data demonstrates the importance of wide surgery in the lumbar spine to reduce the risk of local recurrence and to increase the patients’ survival; not only is this feasible, but the survival rate can be quite comparable to that of the same histologies when the thoracic spine is involved when this highly demanding surgery is performed in specialized centers.

### Limitations of the study

The present study offers a comprehensive overview of EBS for primary malignant bone tumors located in the lumbar spine; however, it is important to note several limitations. Firstly, its retrospective nature exposes it to biases stemming from incomplete or inaccurately reported data. Additionally, being a single-center study, the results may be influenced by the expertise of the senior author and may not fully represent the broader spectrum of cases. The relatively small sample size of 47 patients may not adequately assess rare events occurring in a less common condition.

Furthermore, the long enrollment period from February 1993 to December 2021 introduces potential confounding factors, as advancements in surgical techniques, imaging modalities, and adjuvant therapies could have impacted patient outcomes, thereby complicating the interpretation of results. Despite efforts to gather comprehensive data, there may still be missing information on certain variables, such as patient comorbidities, specific surgical techniques, or long-term functional outcomes.

Lastly, the limited follow-up duration must be acknowledged. Although the study reports a median follow-up of 64.2 months, this may not be sufficient to evaluate the durability of surgical outcomes, recurrence rates, and long-term survival.

## Conclusion

EBS has to be considered a highly demanding surgery, also in the lumbar spine where the complication rate is quite consistent. As already evident for other spine levels [24], treatment should be guided by histological diagnosis to better address possible neoadjuvant therapy and to avoid erroneous approaches. A meticulous surgical plan has to be made to decrease the risk of vascular bundles damage, possible cause of intraoperative death. Wide surgery is still indicated after a first erroneous intralesional approach performed in non-specialized centers, to increase the DFS and the OS.

## Data Availability

The datasets generated and analyzed during the current study are available from the corresponding author on reasonable request.
